# A prospective observational study comparing two supraglottic airway devices in out-of-hospital cardiac arrest

**DOI:** 10.1186/s12873-021-00444-0

**Published:** 2021-04-20

**Authors:** Maja Pålsdatter Lønvik, Odd Eirik Elden, Mats Joakimsen Lunde, Trond Nordseth, Karin Elvenes Bakkelund, Oddvar Uleberg

**Affiliations:** 1 Faculty of Medicine and Health Sciences, Norwegian University of Science and Technology, NO-7491 Trondheim, Norway; 2 Department of Internal Medicine, Nord-Trøndelag Hospital Trust, NO-7601 Levanger, Norway; 3 Department of Emergency Medicine and Pre-Hospital Services, St. Olav’s University Hospital, NO-7030 Trondheim, Norway; 4 Department of Pre-Hospital Services, Nord-Trøndelag Hospital Trust, N-7600 Levanger, Norway; 5 Department of Surgery, Levanger Hospital, Nord-Trøndelag Hospital Trust, N-7600 Levanger, Norway; 6 Department of Circulation and Medical Imaging, Norwegian University of Science and Technology, NO-7491 Trondheim, Norway; 7 Department of Anesthesia and Intensive Care Medicine, St.Olav’s University Hospital, NO-7030 Trondheim, Norway; 8 Department of Research and Development, Norwegian Air Ambulance Foundation, NO-0103 Oslo, Norway

**Keywords:** Airway management, Airway research, Cardiac arrest, OHCA, Emergency medical services, Supraglottic airway device, I-gel, LTS-D, Laryngeal tube, LT, Resuscitation

## Abstract

**Background:**

Airway management in patients with out of hospital cardiac arrest (OHCA) is important and several methods are used. The establishment of a supraglottic airway device (SAD) is a common technique used during OHCA. Two types of SAD are routinely used in Norway; the Kings LTS-D™ and the I-gel®. The aim of this study was to compare the clinical performance of these two devices in terms of difficulty, number of attempts before successful insertion and overall success rate of insertion.

**Methods:**

All adult patients with OHCA, in whom ambulance personnel used a SAD over a one-year period in the ambulance services of Central Norway, were included. After the event, a questionnaire was completed and the personnel responsible for the airway management were interviewed. Primary outcomes were number of attempts until successful insertion, by either same or different ambulance personnel, and the difficulty of insertion graded by easy, medium or hard. Secondary outcomes were reported complications with inserting the SAD’s.

**Results:**

Two hundred and fifty patients were included, of whom 191 received I-gel and 59 received LTS-D. Overall success rate was significantly higher in I-gel (86%) compared to LTS-D (75%, *p* = 0.043). The rates of successful placements were higher when using I-gel compared to LTS-D, and there was a significant increased risk that the insertion of the LTS-D was unsuccessful compared to the I-gel (risk ratio 1.8, p = 0.04). I-gel was assessed to be easy to insert in 80% of the patients, as opposed to LTS-D which was easy to insert in 51% of the patients.

**Conclusions:**

Overall success rate was significantly higher and the difficulty in insertion was significantly lower in the I-gel group compared to the LTS-D in patients with OHCA.

**Supplementary Information:**

The online version contains supplementary material available at 10.1186/s12873-021-00444-0.

## Background

Establishment of a patent airway in order to perform ventilations and chest compressions in addition to early defibrillation are important interventions during the resuscitation of a patient with out-of-hospital cardiac (OHCA) [1]. To establish a patent airway, health care providers commonly use bag-valve-mask ventilation (BVM), or an advanced airway such as supraglottic airway device (SAD) or endotracheal intubation (ETI). Animal studies have shown that a combination of ventilation and chest compressions is more effective than compressions only to preserve oxygenation and to limit hypercapnia [2, 3]. Even though the time spent on ventilation during BVM is not necessarily long, the total hands-off time is significantly longer than the time required for each ventilation [4]. While BVM requires interruptions in compression to perform ventilation, SAD/ETI allows continuous compressions combined with ventilations between compressions. Another advantage is that a fixed airway gives the manual availability for other practical tasks, and no-flow time is reduced when an advanced airway is established [5].

An advantage of the SAD is that it can be inserted without visual inspection, and that it provides a relative air seal tightness around the larynx, which reduces the passing of air into the stomach. Even though some studies show higher survival with the use of ETI compared to SAD [6], SAD is performed quicker and with a higher success rate than ETI when used out of hospital and by less experienced personnel [7–9]. SAD is associated with a lower hands-off time than ETI [10]. European guidelines regarding CPR by ambulance personnel recommends the use of SAD rather than ETI during out of hospital cardiac arrest (OHCA) [1].

Neither the Norwegian nor the European Resuscitation Councils have specific recommendations on the selection of type of SAD, which may explain the use of several different devices among local health trusts [11]. In the three local health trusts in Central Norway two different types are used, the I-gel® (Intersurgical, Wokingham, UK) and the King LTS-D™ (North American Rescue, Greer, SC, US). LTS-D is a laryngeal tube, consisting of a tube with a distal oesophagus cuff and a proximal pharyngeal cuff, which are inflated after insertion in oesophagus. I-gel is an anatomical shaped laryngeal mask, covering all of glottis when placed. Both devices contain a gastric tube port, to decompress the stomach for air or fluids, and thereby reduce the risk of gastric reflux. In our region, ETI is only performed by helicopter emergency medical services, which are manned by anaesthesiologists.

The aim of the study was to investigate the number of attempts before successful insertion and assessment of challenges of insertion in I-gel and LTS-D, when being used by ambulance personnel during out-of-hospital cardiac arrest in Central Norway.

## Methods

The study is a prospective observational study. The study follows the ‘Strengthening the reporting of observational studies in epidemiology’ (STROBE) recommendations for reporting of observational cohort studies [12].

### Study setting

The Central Norway Regional Health Authority has the overall responsibility for the three ambulance services within the health trusts of Møre- og Romsdal (HMR), Nord-Trøndelag (HNT) and St. Olav’s hospital (SOH), covering a total patient population of approximately 721.000 persons. The three ambulance services are separate administrative units, but the Joint Commission of Ambulance Services in Central Norway provide the same guidelines and protocols, and the training and certification of personnel within the three services. The only difference in protocols during this study was the type of SAD used during OHCA. In HMR and SOH the I-gel was used, whereas in HNT the LTS-D was used.

### Data collection

**Fig. 1:**
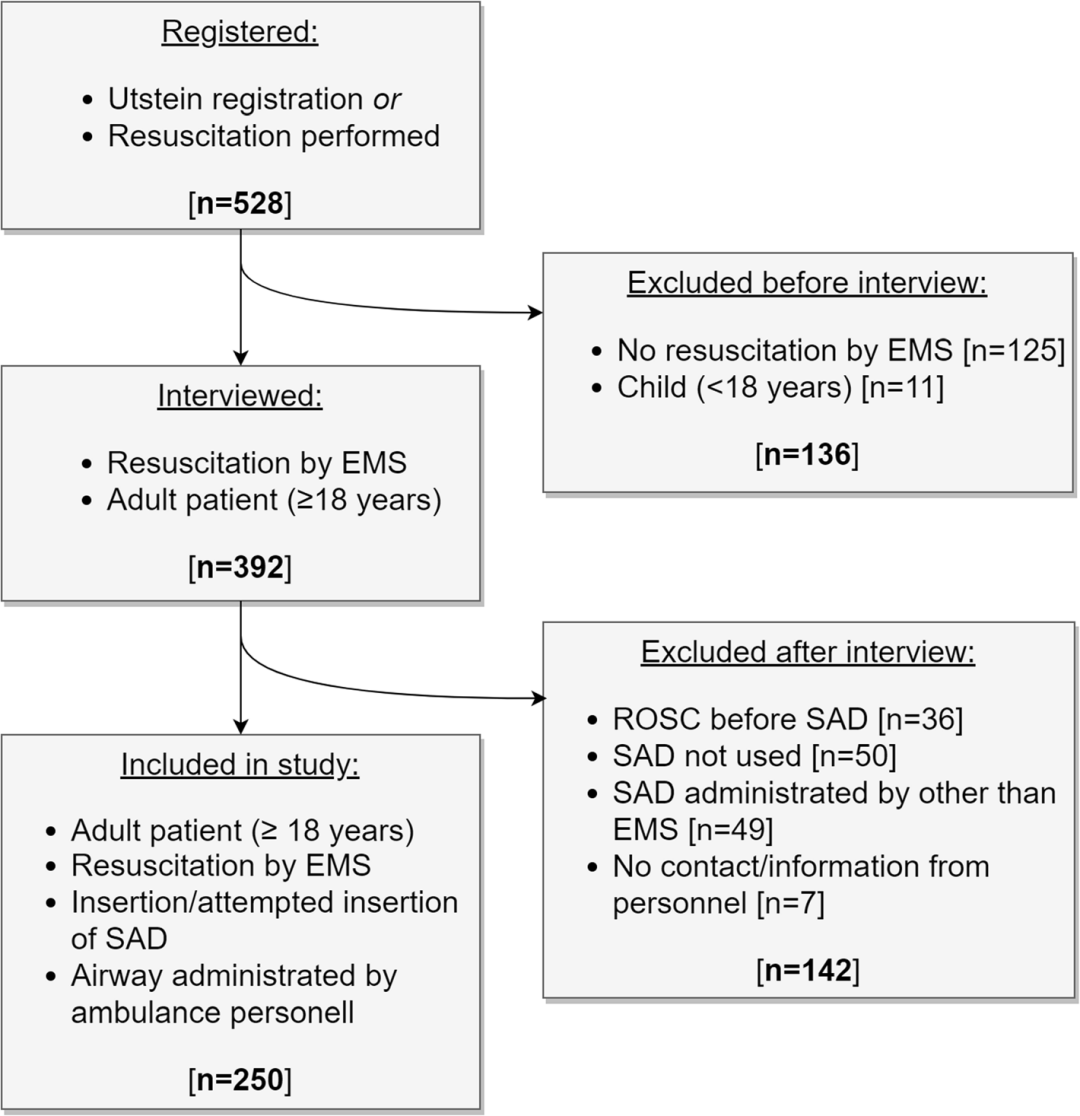
Inclusion and exclusion flowchart. The figure illustrates the numbers and reasons for patient exclusion and the number included patients. Utstein registration and resuscitation attempt definition refer to the Utstein template of uniform reporting for OHCA [13]. **EMS:** Emergency Medical System, **ROSC:** Return of Spontaneous Circulation, **SAD:** Supraglottic Airway Device7

All cardiovascular and / or respiratory arrests in adult patients where ambulance personnel attempted insertion of a SAD during the 12-month period from March 2016 to February 2017 were included. These patients were registered according to the Utstein template of uniform reporting for OHCA and by using the updated definition of a resuscitation attempt: *“the act of trying to maintain or restore life by establishing and/or maintaining breathing and circulation through CPR, defibrillation, and other related emergency care”* [13]. The inclusion process is shown in Fig. 1. Ambulance staff recorded patient data electronically in the electronic patient chart (Ambustat®). Additional study variables were added to further describe characteristics of the performed airway management (Additional file 1). To validate recorded data, every ambulance personnel responsible for airway management was interviewed after every case. This was done to ensure that the form was interpreted and filed correctly. The objective was to do all the interviews within a week after each case, but due to practical reasons, it could take up to a month before the interviews were conducted.

The included OHCA were divided into groups according to which SAD was used. Whenever a need for SAD was identified and the ambulance personnel tried to insert a SAD, it was defined as an attempt. When EMS took the SAD out of the patient’s airway to try a renewed insertion, it was defined as a new attempt. A successful insertion of a SAD was defined as properly positioned and working, as clinically assessed by the ambulance personnel on site. Assessment of proper function was verified by visual confirmation of chest movements, auscultation and/or by the use of capnography. The level of insertion difficulty (easy, medium or hard) were based on the individual perception of the ambulance worker.

### Outcomes

Primary outcomes were number of attempts until successful insertion, by either same or different ambulance personnel, and the difficulty of insertion graded by easy, medium or hard. Secondary outcomes were reported complications with inserting the SAD’s.

### Statistics

Data was analysed in SPSS and R version 4.0.3, applying the R-package “fmsb” [14]. Relevant outcomes were assessed with the Chi-square test, or estimation of risk ratios, as appropriate. The tests were two-sided, and significance was set at *p* <  0.05. Data were reported with 95% confidence intervals when appropriate.

### Ethics

The study was performed in accordance with the Helsinki declaration for medical research involving human subjects. The Regional committees for medical and health research ethics committee of Central Norway (REK - Midt) was informed about the study, and deemed the study a clinical quality study not needing formal Regional Ethics Committee (REC) approval (reference number 2016/127/REC Central). The study received institutional approval (reference number ESA 15/9285) from St. Olav University hospital in Trondheim, which waived the need for patient consent.

## Results

Five-hundred-and-twenty-eight patients with OHCA were treated by the ambulance services, of which two-hundred-and-fifty were included in the study. Baseline patient and event characteristics are presented in Table 1. The total number of excluded patients was 278, in which the main reason for exclusion was no resuscitation efforts started, or airway administration performed by someone other than ambulance personnel (Fig. 1). Seven out of 392 interviews were excluded due to unavailability of adequate information from the ambulance personnel involved. The degree of procedural success and reported difficulty of insertion is demonstrated in Table 2.

**Table 1:** Baseline patient and event characteristics

Patients	I-gel *n* = 191	LTS-D *n* = 59 (%)	p - value
Population in 2017	583,637	137,233	–
Male *n (%)*	138 (72)	44 (75)	0.614
Age *median*	71	70	0.404
ROSC onsite *n (%)*	56 (29)	6 (10)	0.003
30 days survival *n (%)*	27 (14)	1 (2)	0.008

*ROSC* Return of Spontaneous Circulation

**Table 2:** Degree of success and reported difficulty of insertion with estimates of relative risk of applying LTS-D to manage airway compared to applying I-gel

Degree of success	I-gel n = 191 (%)	LTS-D n = 59 (%)	Risk ratio (95% CI)	*p*-value
Successful after 1 or 2 attempts *n (%)*	157 (82)	41 (69)	**0.85** (0.71–1.01)	0.07
Successful after 3 attempts from same personnel *or* Successful after attempts from 2 or more personnel *n (%)*	7 (4)	3 (5)	**1.39** (0.37–5.20)	0.63
Unsuccessful insertion *n (%)*	27 (14)	15 (25)	**1.80** (1.03–3.15)	0.04
Reported difficulty of insertion				
Easy *n (%)*	152 (80)	30 (51)	**0.64** (0.49–0.82)	< 0.001
Medium *n (%)*	24 (13)	13 (22)	**1.75** (0.95–3.22)	0.07
Hard *n (%)*	15 (8)	16 (27)	**3.45** (1.82–6.56)	< 0.001

I-gel was successfully inserted in 86% of the patients, compared to LTS-D, which was successfully inserted in 74% of the patients. The rates of successful placements were higher when using I-gel compared to LTS-D, and there was a significant increased risk that the insertion of the LTS-D was unsuccessful compared to the I-gel (risk ratio 1.80, p = 0.04). I-gel was assessed to be easy to insert in 80% of the patients, as opposed to LTS-D which was easy to insert in 51% of the patients. Difficulty of insertion was significantly higher when using LTS-D compared to I-gel.

### Secondary outcomes

The reported complications are shown in Table 3, but there were no significant differences in the overall amount of complications between the two SADs. Air leakage was more frequently reported in cases where I-gel was used compared to cases where LTS-D was used. Anatomical conditions and problematic insertion were a more frequent challenge in the cases where LTS-D was used compared to cases where I-gel were used.

**Table 3:** Complications of SAD administration

Supraglottic Airway Device	I-gel n = 191 (%)	LTS-D n = 59 (%)	p - value
Any reported complications	92 (48)	32 (54)	0.435
Air-leakage	37 (19)	5 (8)	0.050
Aspiration	24 (13)	8 (14)	0.842
Anatomical conditions	22 (12)	19 (32)	< 0.001
Problematic insertion	13 (7)	10 (17)	0.018
Foreign object	5 (3)	2 (3)	0.753
Hard to ventilate	11 (6)	6 (10)	0.240
Insertion > 30 s	3 (2)	4 (7)	0.034
Bleeding	14 (7)	7 (12)	0.272
Dislocation	11 (6)	2 (3)	0.474
Problems with Bag-Valve-Mask	27 (14)	8 (14)	0.911
Other	14 (7)	4 (7)	0.886

## Discussion

We found that use of the I-gel was associated with a higher success rate and lower complication rate than use of the LTS-D by ambulance personnel during resuscitation from OHCA. The most frequent complication regarding LTS-D was anatomical conditions and problematic insertion, which may explain why the LTS-D may be harder to insert than the I-gel. In our study airway leakage was found to be the most frequent complication regarding use of I-gel.

Endotracheal intubation is the gold standard in prehospital advanced airway management, but requires a level of experience and training often not achievable for ambulance personnel [15]. Several studies show that, at least in hands of less experienced personnel, SAD has a higher insertion success rate and reduced time to secured airway compared to ETI [9, 16, 17]. Skill retention is also high for SAD, showing that less continuous training is needed to adequately use the SAD [16, 18]. When the patient is suboptimal positioned, SAD has an increased success rate compared to ETI, even when being used by skilled anaesthesiologists [19]. During a randomized controlled trial comparing SAD or ETI, fewer of the patients in the ETI group received any advanced airway interventions, which might be associated with SAD being easier to use. In the same study, they noted that among the patients receiving advanced airway management, the patients who received SAD had a higher survival compared to ETI – independent of which group they initially were allocated to [20]. A meta-analysis showed better survival and neurological outcome by the use of ETI compared to SAD, but this included no randomized controlled trials, and did not specify if the personnel using ETI and SAD had the same experience level [6].

In studies comparing I-gel and LTS-D during elective surgery, on cadavers or on manikins most studies are in favour of I-gel when it comes to successful insertions and time to airway control [21–27]. This is consistent with our study results, but it’s important to note that during elective surgery, treatment occurs in a more controlled environment than in the prehospital setting; with more knowledge of the patient in advance, optimal positioning of the patient, optimally anesthetized patient, optimal working height, good lighting, and sufficient personnel present and necessary equipment available. The prehospital setting is often characterized by austere conditions and an unpredictable treatment situation; the lighting conditions may be poor, the space conditions and positioning may be challenging, the patient is unknown, and the personnel resources are limited. The importance of an easy-to-use airway management tool is essential, as it is necessary to share the attention between airway management and other work tasks. We have not found any studies comparing the two SADs in out-of-hospital cardiac arrests.

We recognize some limitations in our study. First, the type of airway device used depended on which ambulance service the ambulance personnel belonged to, thus there was two groups with I-gel and one with LTS-D. Second, the three separate services had the same certification requirements and monthly case training on cardiac arrest situations and airway administration, but one would expect that educational motivation and updating would differ a little between ambulance bases in the same service. Third, the level of competence and experience of the ambulance personnel being responsible for each airway administration was not recorded in this study. All personnel have completed training in supraglottic airway administration, but personal education and experience may differ based on years in service and the local incidence of OHCA. This was not further investigated, which cannot exclude the possibility of results being affected by differences in overall competence in the three ambulance services. Fourth, the population density differs within the three health trusts. In services close to the cities, in Trondheim in particular, the population density is significantly larger than in the rural areas. This may affect the experience, and thus the competence of the individual ambulance personnel. Differences in geography may also affect ambulance response times, which may affect the probability of survival after cardiac arrest. Extended use of time before the initiation of resuscitation reduces the likelihood of survival [28]. However, we assumed that this would not affect the primary endpoints. Anatomical conditions were a reported problem in a third of the cases where LTS-D was used, but we have no reason to believe there is any population based differences between the two groups regarding anatomical conditions.

Despite some limitations, the data in this study reflect real-life situations and how the two different devices perform in pre-hospital clinical services. There are several environmental differences affecting practice when comparing the pre- and in-hospital setting. Results from previous studies performed in the in-hospital setting (i.e. operating room) might therefore not be directly transferrable to the pre-hospital setting. By conducting a telephone interview of all involved ambulance workers, the quality data could be validated. As an example, several ambulance personnel did initially not register the use of SAD as an airway intervention if they did not achieve a successful insertion. Without a telephone interview, the number of missed interventions would potentially have been substantially higher.

## Conclusions

The study showed a difference between the use of LTS-D and I-gel during OHCA in the clinical setting of the ambulance service in Central Norway. Overall success rate was significantly higher and the difficulty in insertion was significantly lower in the I-gel group compared to the LTS-D group. The use of I-gel during OHCA is associated with both being easier and more often successfully inserted when compared with LTS-D.

## Supplementary Information

**Additional file 1.**

## Abbreviations

OHCA: Out of Hospital Cardiac Arrest
ROSC: Return of spontaneous circulation
SAD: Supraglottic Airway Device
ETI: Endotracheal intubation
BVM: Bag-valve-mask ventilation
CPR: Cardiopulmonary Resuscitation
HNT: Nord-Trøndelag Hospital Trust
HMR: Møre- og Romsdal Hospital Trust
SOH: St. Olav’s University Hospital
CI: Confidence Interval
STROBE: Strengthening the reporting of observational studies in epidemiology
REC: Regional Committee for Medical and Health Research Ethics

## Declarations

### Availability of data and material

The datasets used during the current study are available from the corresponding author on reasonable request.
