# Supplementary material for: A prospective observational study comparing two supraglottic airway devices in out-of-hospital cardiac arrest
Source: BMC Emerg Med. 2021 Apr 20;21:51. doi: 10.1186/s12873-021-00444-0 (PMC8056505; doi:10.1186/s12873-021-00444-0)
Supplement: Supplementary file 1 — Additional file 1. [file 12873_2021_444_MOESM1_ESM.docx]

**A prospective observational study comparing two supraglottic airway devices in out-of-hospital cardiac arrest.**

Maja Pålsdatter Lønvik ^1,2^, Odd Eirik Elden­­ ^3,4,5^, Mats Joakimsen Lunde ^5^, Trond Nordseth ^6,7^, Karin Elvenes Bakkelund ^3^, Oddvar Uleberg ^3,8^

1. Faculty of Medicine and Health Sciences, Norwegian University of Science and Technology, NO-7491 Trondheim, Norway
2. Department of Internal Medicine, Nord-Trøndelag Hospital Trust, NO-7601 Levanger, Norway
3. Department of Emergency Medicine and Pre-Hospital Services, St. Olav’s University Hospital, NO-7030 Trondheim, Norway
4. Department of Pre-Hospital Services, Nord-Trøndelag Hospital Trust, N-7600 Levanger, Norway
5. Department of Surgery, Levanger Hospital, Nord-Trøndelag Hospital Trust, N-7600 Levanger, Norway
6. Department of Circulation and Medical Imaging, Norwegian University of Science and Technology, NO-7491 Trondheim, Norway.
7. Department of Anesthesia and Intensive Care Medicine, St.Olav’s University Hospital , NO-7030 Trondheim, Norway
8. Department of Research and Development, Norwegian Air Ambulance Foundation, NO-0103 Oslo, Norway

Maja Pålsdatter Lønvik: [lonvikm@hotmail.com](mailto:lonvikm@hotmail.com)

Odd Eirik Elden: [oddeirik.elden@helse-nordtrondelag.no](mailto:oddeirik.elden@helse-nordtrondelag.no)

Mats Joakimsen Lunde: [mats.lunde@gmail.com](mailto:mats.lunde@gmail.com)

Trond Nordseth: [trond.nordseth@ntnu.no](mailto:trond.nordseth@ntnu.no)

Karin Elvenes Bakkelund: [karin.bakkelund@stolav.no](mailto:karin.bakkelund@stolav.no)

Oddvar Uleberg: [oddvar.uleberg@stolav.no](mailto:oddvar.uleberg@stolav.no)

**Corresponding author:**

Oddvar Uleberg, MD PhD

Department of Emergency Medicine and Pre-Hospital Services, St. Olav’s University Hospital, NO-7030 Trondheim, Norway

Tel. +47 482 66 455

**Additional file 1 - Variables registered**

| gender and age of patient |
| --- |
| which health trust the ambulance personnel belonged to |
| which airway device were used |
| the number of attempts before SAD was successful placed |
| how easy it was to put down SAD |
| problems / complications with the procedure   - - air leak   - vomiting / aspiration   - anatomical conditions (wheezing / difficult mouth opening / stiff neck)   - problematic placement (“gores”, must be rotated down)   - foreign object   - hard to ventilate   - Placement > 30 seconds   - bleeding in the oral cavity / respiratory tract   - The tube dislocates during resuscitation or transport   - Problems with bag-valve-mask ventilation   - Other |
| time from start CPR to successfully located SAD |
| outcomes and survival |
